# The Anti-Inflammatory Compound Curcumin Enhances Locomotor and Sensory Recovery after Spinal Cord Injury in Rats by Immunomodulation

**DOI:** 10.3390/ijms17010049

**Published:** 2015-12-31

**Authors:** Lucia Machova Urdzikova, Kristyna Karova, Jiri Ruzicka, Anna Kloudova, Craig Shannon, Jana Dubisova, Raj Murali, Sarka Kubinova, Eva Sykova, Meena Jhanwar-Uniyal, Pavla Jendelova

**Affiliations:** 1 Institute of Experimental Medicine, Academy of Sciences of the Czech Republic, Vídeňská 1083, Prague 142 20, Czech Republic; urdzikl@gmail.com (L.M.U.); karova@biomed.cas.cz (K.K.); j.ruzicka@biomed.cas.cz (J.R.); anna.kloudova@gmail.com (A.K.), jana.dubisova@biomed.cas.cz (J.D.); sarka.k@biomed.cas.cz (S.K.); sykova@biomed.cas.cz (E.S.); 2 Department of Neuroscience, Charles University, Second Faculty of Medicine, Prague 142 20, Czech Republic; 3 New York Medical College, New York, NY 10595, USA; cshannonmd@gmail.com (C.S.); Raj_Murali@NYMC.edu (R.M.); meena_jhanwar@NYMC.edu (M.J.-U.)

**Keywords:** spinal cord injury, curcumin, inflammation, cytokines, secondary processes, NF-κB

## Abstract

Well known for its anti-oxidative and anti-inflammation properties, curcumin is a polyphenol found in the rhizome of *Curcuma longa*. In this study, we evaluated the effects of curcumin on behavioral recovery, glial scar formation, tissue preservation, axonal sprouting, and inflammation after spinal cord injury (SCI) in male Wistar rats. The rats were randomized into two groups following a balloon compression injury at the level of T9–T10 of the spinal cord, namely vehicle- or curcumin-treated. Curcumin was applied locally on the surface of the injured spinal cord immediately following injury and then given intraperitoneally daily; the control rats were treated with vehicle in the same manner. Curcumin treatment improved behavioral recovery within the first week following SCI as evidenced by improved Basso, Beattie, and Bresnahan (BBB) test and plantar scores, representing locomotor and sensory performance, respectively. Furthermore, curcumin treatment decreased glial scar formation by decreasing the levels of MIP1α, IL-2, and RANTES production and by decreasing NF-κB activity. These results, therefore, demonstrate that curcumin has a profound anti-inflammatory therapeutic potential in the treatment of spinal cord injury, especially when given immediately after the injury.

## 1. Introduction

Spinal cord injury (SCI) is a devastating medical condition that can temporarily or permanently impair sensory and motor functions. SCI consists of a two-step process involving the initial physical injury leading to a progressive injury process (secondary injury) that comprises glial scar formation, inflammation, lipid peroxidation, and glutamate excitotoxicity. The initial physical injury, creating mechanical damage to the spinal cord, leads to tissue necrosis and the disruption of neuronal and vascular structures. However, secondary processes that result in glial scar formation and hinder recovery evolve within hours to days. Many studies in recent years have investigated a variety of pharmacological interventions targeting these secondary processes, such as methylprednisolone, melatonin, erythropoietin, and naloxone, all of which resulted in only a slight improvement of impaired function after SCI. Curcumin (diferuloylmethane) [1,7-bis(4-hydroxy-3-methoxyphenyl)-1,6-heptadiene-3,5-dione] is responsible for turmeric’s yellow color (the ground rhizome of *Curcuma longa* L.), which is commonly used as a spice but is also known for its potent anti-inflammatory properties. As previously reported, curcumin influences the NF-κB pathway, which is involved in proinflammatory cytokine production, antiapoptotic processes, the recruitment of leukocytes, and cell survival, all of which are important contributors to the inflammatory response [1]. Studies have shown that administering curcumin can improve SCI [2], but the exact mechanisms that lead to better recovery have not been fully brought to light [3].

The present study was designed to investigate the effect of curcumin application in the treatment of SCI in rats during lesion development. Behavioral performance, glial scar formation, tissue preservation, axonal sprouting, the activity of NF-κB transcription factor, and the levels of proinflammatory cytokines were evaluated after experimental SCI to elucidate the mechanisms underlying the effect of curcumin on spinal cord lesion development.

## 2. Results

### 2.1. Behavioral Assessment

#### 2.1.1. Basso, Beattie, and Bresnahan (BBB) Test

**Figure 1:**
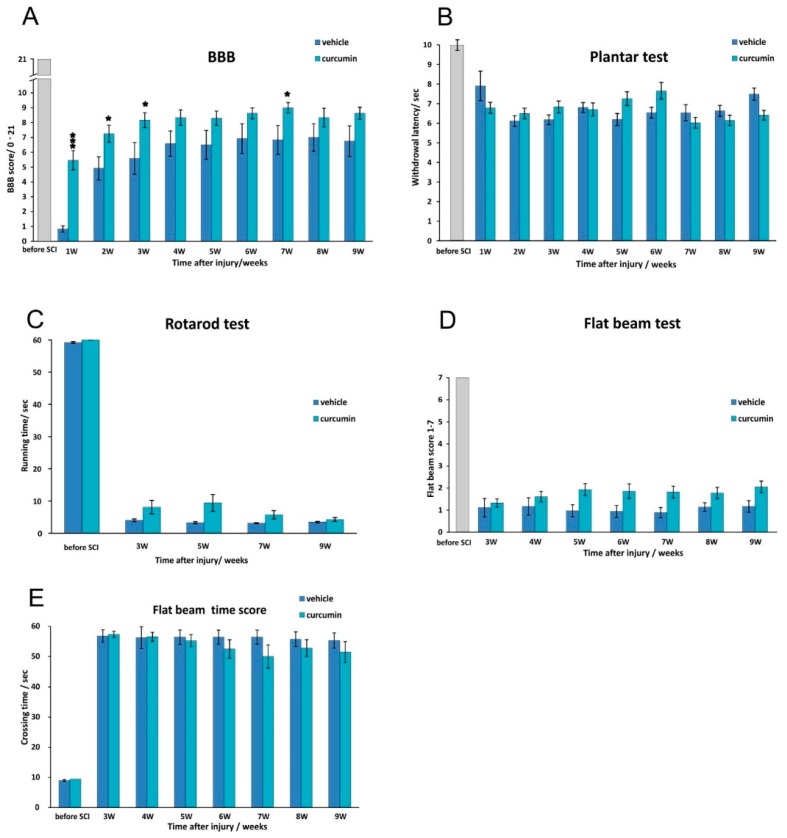
Functional recovery after SCI: The BBB locomotor scale in control and curcumin-treated animals (**A**); Statistically significant differences were observed 1, 2, 3, and 7 weeks after curcumin treatment; we used the plantar test to measure the effect of curcumin on thermal nociception (**B**); We observed that curcumin had no significant effect on hyperalgesia; the rotarod device tested the ability of the rats to balance on a rotating pole (**C**); the flat beam test was used to evaluate muscle strength and forelimb-hindlimb coordination in both the control and curcumin groups (**D**); The curcumin-treated group performed better on the flat beam test throughout the whole study period, but the differences were not statistically significant. The flat beam time score reflects the ability of the animals to cross the beam within a maximum of 60 s; no significant differences between the groups were observed (**E**); * *p* < 0.05, and *** *p* < 0.001 indicate statistical significance.

An open field evaluation of locomotor recovery after SCI was conducted using the BBB locomotor rating scale [4]. Rats treated with curcumin displayed significantly better locomotor recovery at the early stages of treatment when compared to control rats and also reached a significantly higher BBB score than did the vehicle-treated animals. At two and three weeks after injury, curcumin-treated rats still performed significantly better than the control rats, but the difference between the groups was narrower than in the first week. At weeks 4, 5, and 6, curcumin-treated rats had better BBB scores than the controls, but the differences were insignificant. Statistically significant differences in the BBB scores was again observed at week 7, when the curcumin-treated rats had a better score (Figure 1A).

#### 2.1.2. Plantar Test

The plantar test was used to determine thermal nociception (hyperalgesia) after SCI in both control and curcumin-treated rats. Starting a week after injury, we observed no significant difference between the control and curcumin-treated groups. At 5 and 6 weeks after injury, the withdrawal latency slightly increased in the curcumin group, but the increase was not statistically significant (Figure 1B).

#### 2.1.3. Rotarod Test

The rotarod test evaluates the ability of an animal to balance on a rotating pole. Rats with curcumin treatment were able to balance on a rotating pole longer than control rats at the 3rd, 5th and 7th weeks after SCI, but the difference between the two groups was not significant (Figure 1C).

#### 2.1.4. Flat Beam Test

The flat beam test was used to measure the recovery of motor function and forelimb–hindlimb coordination. The balloon compression lesion produced submaximal SCI, and the majority of the rats started with no or only limited ability to balance on the beam while stationary. The curcumin-treated group performed better on the flat beam test during the entire course of the study; however, the differences between the groups remained statistically insignificant (Figure 1D). The flat beam time score reflects the time required for the animals to move across the beam within a maximum of 60 s. Rats treated with curcumin were faster in crossing the beam, but the overall difference between the two groups was insignificant during the entire study period (Figure 1E).

### 2.2. Histology and Immunohistochemistry

#### 2.2.1. NF-κB Activity

**Figure 2:**
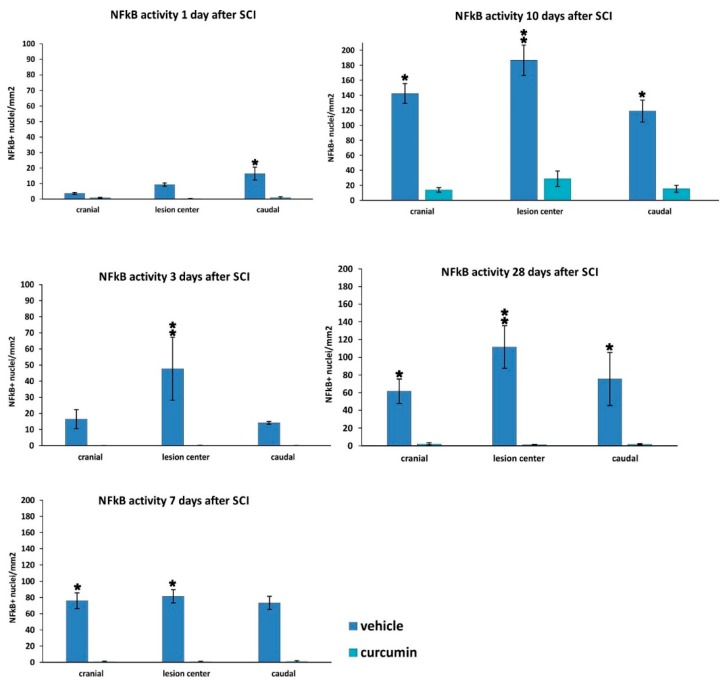
Measurement of NF-κB activity 1, 3, 7, 10 and 28 days after SCI in control and curcumin-treated rats. The graphs depict a significant decrease in NF-κB activity, as measured by its nuclear translocation, in the central, cranial and caudal parts of the spinal cord lesion after curcumin treatment. * *p* < 0.05, ** *p* < 0.01 indicate statistical significance.

The numerical density of NF-κB (p65)-positive nuclei was evaluated on transversal spinal cord sections 1, 3, 7, 10, and 28 days post-SCI in the curcumin- and vehicle-treated animals (Figure 2). The translocation of NF-κB into the nuclei increased during SCI development. The highest number of NF-κB-positive nuclei was observed at 10 days after SCI, followed by a slight decrease. Curcumin treatment led to a significant reduction in the density of NF-κB-positive nuclei in all treated rats at all time points when compared to control rats with the exception of 1 day after SCI, when only a local reduction was seen caudally from the lesion center. At the other time points, the curcumin-treated rats displayed a marked reduction in NF-κB activity, especially in the central parts of the injury. At the later time points (10 and 28 days post-SCI), the translocation of p65 into the nucleus was prevented by curcumin treatment in all evaluated areas of the spinal cord tissue.

#### 2.2.2. White and Grey Matter Sparing

**Figure 3:**
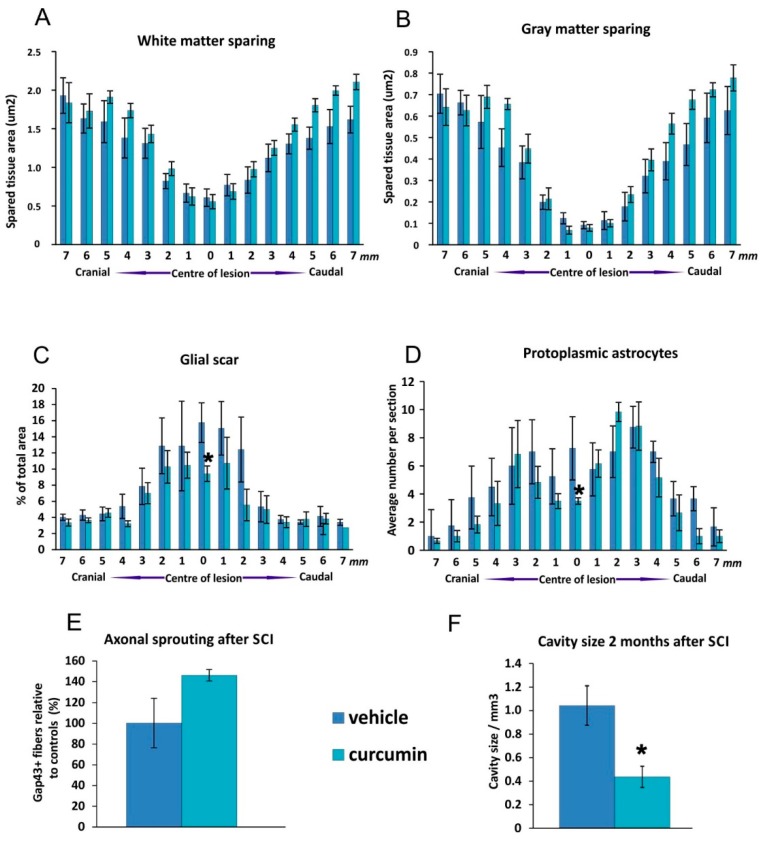
Morphometry measurements of the extent of spared white and gray matter in the center of the spinal cord lesion (**A**,**B**); Compared to the control group, the white matter was not significantly more preserved in the curcumin-treated group 4–5 mm caudally and 5–7 mm cranially from the center of the lesion. The gray matter in the curcumin-treated group was more preserved 4–5 mm caudally and 4–7 mm cranially from the spinal cord lesion when compared to controls but this was statistically insignificant. Significant changes in the extent of the GFAP-positive area expressed as a percentage of the total area of the tissue slice after curcumin treatment (**C**); We found a higher number of protoplasmic astrocytes in the central part of the spinal lesion in control animals when compared to the curcumin-treated rats (**D**); The effect of curcumin application on axonal sprouting two months after injury. No significant differences were observed when compared to the control group (**E**); The volume of cavities in curcumin treated SCI tissue was significantly smaller than in control spinal cord tissue (**F**). Statistical significance was marked with *, where *p* < 0.05.

Nine weeks after SCI we measured the extent of spared white and gray matter on transversal spinal sections (Figure 3). The white matter was more preserved in the curcumin-treated group at 4–5 mm caudally and 5–7 mm cranially from the lesion center compared to the control group (Figure 3A). The gray matter in the curcumin-treated group was also more preserved 4–5 mm caudally and 4–7 mm cranially from the spinal cord lesion when compared to controls (Figure 3B). The volume of the cavities in the spinal cord tissue was smaller after curcumin treatment compared to controls. (Figure 3F).

#### 2.2.3. Glial Scar Re-Modulation

The extent of GFAP (glial fibrillary acidic protein) positive area was measured two months post-injury (Figure 3C). The distribution of glial scarring was similar in both groups in the peripheral region of spinal cord while significantly stronger positivity was seen around the central part of the lesion area in control animals when compared to curcumin-treated rats. Also, the number of protoplasmic astrocytes was significantly higher around the center of the control group’s lesion in comparison to the curcumin-treated rats (Figure 3D).

#### 2.2.4. Axonal Sprouting

GAP43-stained transversal spinal sections were used to determine the number of newly sprouted axonal fibers two months after injury. We observed no significant difference in the mean number of GAP43-positive fibers per slice between the curcumin-treated and control rats (Figure 3E).

### 2.3. Inflammatory Cytokines

The levels of secreted inflammatory cytokines were measured using a customized Milliplex inflammatory cytokine kit (Millipore, Billerica, MA, USA) and Magpix instrumentation software MILLIPLEX (Millipore, Billerica, MA, USA).

**Figure 4:**
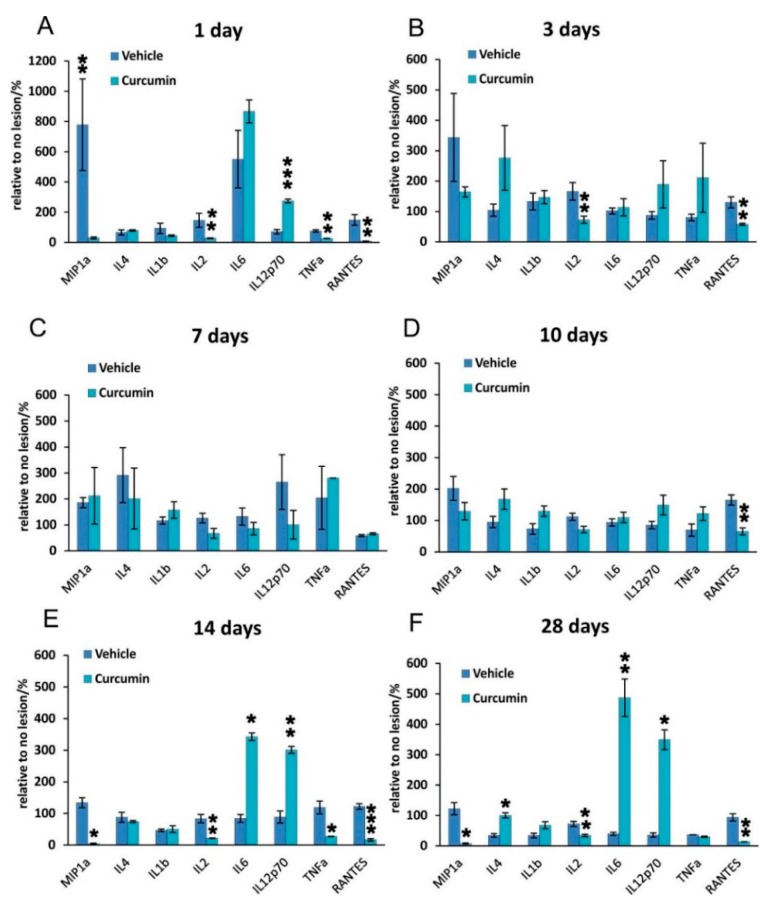
Levels of proinflammatory cytokines 1, 3, 7, 10, 14, and 28 days after SCI in curcumin- and vehicle-treated rats. The levels of IL-2 were significantly lower in the curcumin-treated group compared to vehicle treatment 1 and 3 days after SCI (**A**,**B**); The levels of cytokines 7 and 10 days after SCI were not significantly influenced by curcumin treatment with the exception of RANTES, which was more abundant in control spinal cords 1, 3, 10, 14, and 28 days after SCI (**C**,**D**); The levels of TNF-α reached statistical significance in curcumin-treated spinal cords compared to controls 1 and 14 days after induction of the lesion. IL-2 again significantly decreased 14 and 28 days after injury in the curcumin group (**E**,**F**). IL-6 and IL-12p 70 were upregulated by curcumin treatment on day 1 and then 14 and 28 days post-lesion. MIP-1α reached significantly higher levels in the control group 1, 14 and 28 days after SCI. * *p* < 0.05, ** *p* < 0.01, and *** *p* < 0.001 indicates statistical significance.

When comparing the curcumin and control groups across the entire time span of treatment (1–28 days), we found significant changes in the levels of these cytokines MIP-1α, IL-2, IL-6, IL-12 p70, and RANTES (*p* ˂ 0.001, two way ANOVA). The evaluation of individual cytokines at specific time points revealed changes in the levels of IL-4, IL-1β, IL-2, IL-6, IL-12, TNF-α, MIP-1α, and RANTES in the lesioned area when evaluated 1, 3, 7, 10, 14, and 28 days after SCI (Figure 4). In the curcumin-treated rats, the levels of IL-2 were significantly lower than in untreated animals 1 and 3 days after SCI and then again 14 and 28 days after the injury. On days 7 and 10, the IL-2 levels were lower; however, statistical significance was not reached. The levels of TNF-α reached statistical significance in the spinal cords of curcumin-treated animals compared to controls 1 day and 14 days after the induction of the lesion. In contrast, RANTES and MIP-1α were more abundant in control spinal cords at the beginning and the end of the 28-day observation period. The curcumin group showed significantly higher levels of IL-6 and IL-12 p70 than the vehicle-treated controls 14 and 28 days after SCI. The curcumin-treated group showed significantly higher levels of IL-4 on the 28th day following SCI.

### 2.4. Gene Expression

**Figure 5:**
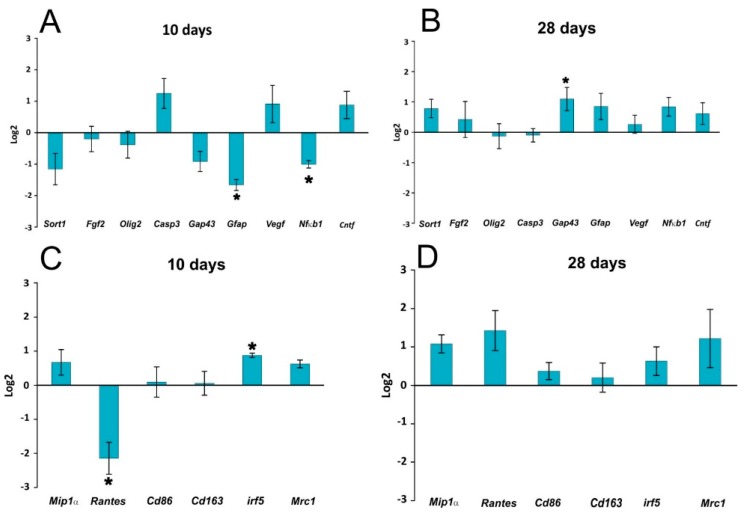
Gene expression profiling in the lesion center 10 and 28 days following SCI. The graphs show genes related to regenerative processes (growth factors, apoptosis, axonal sprouting, astrogliosis) (**A**,**B**) and genes related to the immune response (proinflammatory cytokines and M1 and M2 macrophages); (**C**,**D**).The graphs show the log2-fold changes of the indicated genes 10 and 28 days after curcumin treatment in comparison to the control values. The data is shown as the mean ± SEM, * *p* < 0.05 (ΔΔ*C*_t_ values of curcumin *vs.* vehicle).

The expressions of genes related to the immune response and inflammation (*Mip1α*, *Rantes*, *Cd86*, *Cd163*, *Irf5*, *Mrc1*, and *Nfkb1*), vascularization (*Vegfa*), growth factors (*Sort1*, *Fgf2*, *Cntf*), axonal sprouting (*Gap43*), astrogliosis (*Gfap*), and oligodendrocytes (*Olig2*) was observed 10 and 28 days after lesion induction (Figure 5). The expression of these genes was not markedly changed after curcumin treatment. A significant decrease of mRNA expression was found for GFAP at 10 days, while on the other hand *Irf5* expression was upregulated at 10 days and *Gap43* at 28 days. The decreased expression of *Gfap* correlated with the histological findings, where a significantly smaller GFAP positive area was found in the curcumin-treated rats. Similarly, the downregulation of RANTES mRNA and NF-κB mRNA levels corresponded to the results of the cytokine analysis and, for NF-κB, also to the immunohistochemical staining of NF-κB-positive nuclei.

## 3. Discussion

Our study demonstrated that the anti-inflammatory compound curcumin enhanced recovery after an experimental spinal cord lesion in rats in comparison with vehicle treatment. Histopathological investigation showed that glial scar formation was reduced after curcumin treatment in the lesion, as was confirmed by the expression of the *Gfap* gene 10 days after SCI. The levels of pro-inflammatory cytokines were also reduced by curcumin treatment. 

The primary mechanical insult to the spinal cord leads to several secondary mechanisms that cause progressive central cavitation and glial scar formation [5]. Inflammation is one of the main secondary mechanisms that damage the spinal tissue at molecular, cellular, and neural levels. Increased levels of inflammation-related factors within the injured spinal cord, including TNF-α, IL-1β, IL-6 and their mediator NF-κB, are believed to contribute to the spinal cord damage [6,7]. It has been documented that curcumin exerts its anti-inflammatory effect via the inhibition of molecules involved in inflammation [8]. TNF-α is a major mediator of inflammation in most diseases, and its effect is regulated by the activation of the nuclear factor NF-κB signaling pathway. However, there are a number of other inflammatory mediators that are regulated by NF-κB, including inflammatory cytokines, chemokines, adhesion molecules, enzymes, and kinases [8,9,10]. It has been shown that curcumin is an effective inibitor of NF-κB activation induced by different inflammatory stimuli [11]. This effect was also confirmed in our study showing that the application of curcumin after SCI inhibits the NF-κB signaling pathway. Curcumin is also a potent suppressor of TNF-α; in our experiments, we observed a significant suppression of TNF-α levels on days 1 and 14. The reduction of TNF-α levels on day 1 may be important in curcumin’s effect on TNF-α related pathways and the reduction of inflammation during the early stage of SCI. Most likely, the effect of curcumin during the early stage of SCI correlates with an improved BBB score during the first week after SCI. The transient increase of TNF-α between days 3 and 14 might be due to the migration, activation, and accumulation of immune response cells (neutrophils, macroglia, T-cells and macrophages) during this period. The effect of curcumin might not be strong enough or may not persist long enough to decrease the levels of TNF-α released from these inflammatory cells. After the numbers of immune response cells stabilize, the level of TNF-α decreases. Future detailed studies could define the precise mechanism responsible for these findings.

More importantly, curcumin reduces the inflammatory cascade and thereby aids in preventing plaque deposition and protein oxidation under neurodegenerative conditions. Curcumin has been shown to target various important transcription factors and enzymes including NF-κB, HIF-1α, chemokine receptor CXCR4, and MMP-9 [12]. Due to these properties, curcumin can reduce secondary injury to the spinal cord and thus facilitate better functional recovery [13]. In recent years, numerous studies have established that curcumin’s mechanism of action is via the suppression of NF-κB signaling pathways activated during inflammation and the inhibition of interleukin IL-6-induced STAT3 phosphorylation and consequent STAT3 nuclear translocation [8,14,15,16,17]. Curcumin also reduces the production of inducible nitric oxide synthase and cyclooxygenase (COX)-2 mRNA due to limiting activator protein AP-1 and nuclear factor NF-κB-mediated gene transcription [18]. Curcumin exerts a neuroprotective effect by its activation of the wnt signaling pathway [19]. In traumatic brain injury, curcumin reduces the acute activation of microglia and neuronal apoptosis through a mechanism involving the TLR4/MyD88/NF-κB signaling pathway [20]. Curcumin treatment increases the expression of transcription factor Nrf-2, which acts as a transcriptional regulator of antioxidant and detoxifying enzymes [21].

IL-2 exerts its actions by binding to various forms of the IL-2 receptor (IL-2R), and this association triggers multiple signaling pathways, such as Jak/Stat, PI3K/Akt and MAPK, to generate immune responses [22]. The proinflammatory cytokine IL-2 is secreted by several types of immune cells including T cell receptor (TCR) αβ+ and TCRγδ+ T cells, natural killer (NK) cells, NKT cells, dendritic cells, and mast cells. The activation of IL-2 is generally seen as an immune response. It our study, we observed that IL-2 levels were lower after curcumin treatment at all time points, suggesting that IL-2-related immune functions were also suppressed.

We observed a very different response of IL-6 after curcumin treatment. IL-6 is a multifunctional cytokine that plays important roles in the inflammatory response in many tissues. Recent studies have shown that curcumin is a potent inhibitor of IL-6 [23]. In our study, we observed that IL-6 levels were not altered on days 1, 3, 7 and 10 but were increased on days 14 and 28. This is perhaps due to the fact that curcumin has the greatest effect in the early stages of SCI or that it exerts its effects only for a limited period of time.

We observed that the BBB score improved within one week of curcumin treatment (Figure 1). Thereafter, the improvement persisted for a few weeks before reaching a plateau, but the extent of improvement remained higher than that seen in the controls; the difference between the two groups reached significance again later in the recovery process (week 7). Those observations correlate with the time course of the immunomodulatory effect of curcumin based on our cytokine analysis. Interestingly, curcumin remained less effective in improving the results of behavior tests that required more complex movement patterns by the rat (Figure 1).

In our study, curcumin reduced glial scar formation and suppressed the expression of GFAP mRNA, which may contribute to a more favorable environment for SCI repair. The observations of Wang *et al.* [24] showing a reduction of GFAP expression by Western blot analysis are in accordance with our results. Cytokine analysis of the curcumin-treated rats revealed a noticeable reduction of MIP-1α, IL-2 and RANTES on day 1, while the level of IL-12p70 increased on day 1. TNF-α was shown to be reduced by curcumin on days 1 and 14 of treatment. Similarly, a recent study has shown that curcumin treatment significantly reduced the elevated levels of the pro-inflammatory cytokine TNF-α and NF-κB expression generated by SCI within 24 h of treatment [25]. Curcumin treatment significantly decreased the expression of nuclear NF-κB (Figure 2) and lowered the activity of the NF-kB pathway, as also confirmed by gene expression analysis (Figure 5). However, in our study, NF-κB activity did not correlate solely with a decrease of TNF-α, since NF-κB, unlike TNF-α, was reduced at all time points. A decrease of other cytokines (IL-2 and RANTES) together with a transient decrease of TNF-α can lead to the decreased production of chemokines by both astrocytes and microglia, thus we can expect less infiltration by macrophages and other cells of the immune system, which in turn reduces the elevated levels of various cytokines at the site of the injury [26,27]. The major action of curcumin does not occur only via the inhibition of TNF-α production and NF-κB activity. Moreover, the anti-inflammatory activity of curcumin is shown by its suppression of MAPK pathways in addition to that of NF-κB, as seen in ischemic brain diseases [28].

The anti-inflammatory action of curcumin led to an improvement of the BBB score in the first weeks after SCI, but the effect was not sufficient to prevent the development of secondary injury in the spinal lesion, and in the later phase of the injury the effect on the BBB score and morphometrical measurements diminished. The neuroprotective effect of curcumin could be potentiated by being combined with long-term therapies such as the implantation of biomaterials or neural precursors [3]. A multifactorial approach is essential to support the regeneration and repair of spinal cord tissue initiated by curcumin in the early stages of spinal cord lesion development. 

## 4. Experimental Section

### 4.1. Spinal Cord Injury

Ten-week-old male Wistar rats (obtained from the breeding facility of the Institute of Physiology, Academy of Sciences of the Czech Republic, Prague, Czech Republic) were used in the study. Their body weight was in the range of 300 ± 15 g to keep body size differences to a minimum and thus achieve standardized spinal cord lesions. Based on our previous studies, the number of animals was statistically optimized for each particular experiment to reduce the total number of animals used according to European Commission Directive 2010/63/EU.

A balloon-induced compression lesion was used as an experimental model of SCI in rats [29]. After administering isoflurane anesthesia, a Fogarty catheter (2 French) was inserted into the epidural space through a laminectomy at the T10 vertebral arch cranially, positioning the center of the balloon at the T8 spinal level. Body temperature was kept a constant 37 °C during the entire surgical procedure. Spinal cord compression was induced by inflating the balloon with 15 µL of saline for 5 min. As soon as the lesion was induced, we removed the catheter and sutured the wound in anatomical layers (muscles, subcutaneous tissue, and skin). Gentamicin (Lek Pharmaceutical, Ljubljana, Slovenia, 5 mg/kg) was given intramuscularly to all groups of animals to prevent post-surgical and urinary tract infections. After lesioning, all rats were affected by urine retention, so in most cases bladders were relieved manually twice per day for 2 to 3 weeks. 

Immediately after SCI, the rats were divided into two groups at random. The first group was subject to intraperitoneal curcumin treatment daily (Curcumin, Sigma-Aldrich, St. Louis, MO, USA, 6 mg/kg diluted in olive oil) from 1–28 days after SCI and weekly *in situ* on the 1, 7, 14, 21 and 28 days post-lesion (Curcumin, Sigma, St. Louis, MO, USA, 60 mg/kg/mL diluted in olive oil). The second group received vehicle (olive oil) on the same days as the first group. The timing and doses were chosen according to the study of Ormond *et al.* [3].

The rats were utilized in two sets of experiments; the first set of experiments consisted of cytokine detection and qPCR evaluation, while the second set of animals survived for 9 weeks and was used for behavioral assessment and for histological and immunohistochemical analyses. The animals used for the cytokine experiments were sacrificed 1, 3, 7, 10, 14, or 28 days after injury, five rats at each time point from each group, *i.e.*, a total of 30 rats with curcumin treatment and 30 rats as controls. Twelve rats with curcumin treatment and twelve rats treated with vehicle were used for behavioral assessment and for histological and immunohistochemical analyses and they were sacrificed after nine weeks. All animals were kept on a 12:12 light/dark cycle with *ad libitum* access to food and water.

All experiments were performed in accordance with the European Communities Council Directive of 22 September 2010 (2010/63/EU) regarding the use of animals in research and were approved by the Ethics Committee of the Institute of Experimental Medicine, Academy of Sciences of the Czech Republic, in Prague.

### 4.2. Behavioral Assessment

Functional testing was performed before SCI and then weekly thereafter. The rats were pre-trained for each particular behavioral test to adapt to the testing conditions. 

#### 4.2.1. BBB Test

Locomotor activity was appraised weekly using the Basso, Beattie, and Bresnahan test [4]. The rat was placed in a large open arena, and two independent examiners studied its locomotor ability for 4 min once a week, starting the first week after injury. The BBB test is designed to describe hindlimb joint movement, paw placement, body weight support, forelimb-hindlimb coordination and other parameters according to a scale from 0–21. Each limb was evaluated separately, but averaged in the final analysis.

#### 4.2.2. Flat Beam Test

The flat beam test was used to evaluate motor function and forelimb-hindlimb coordination. The apparatus consisted of a 3.4-cm-wide and 140-cm-long rectangular wooden beam. A goal box was placed at one end. For evaluating the walking distance, only the central 1 m long part of the beam was used. A video-tracking system (TSE-Systems Inc., Bad Homburg, Germany) recorded and evaluated the latency and the trajectory of the rat traversing the beam for a maximum of 60 s. This test was conducted twice a day for three consecutive days. The animals were pre-trained and examined before surgery, then examined again weekly starting the second week after lesion induction. A modified Goldstein scale [30] was used to quantify locomotor function. The rats were scored on a scale from 0–7, ranging from no ability to balance on the beam to crossing the whole length of the beam properly using both hindlimbs (Supplementary Material).

#### 4.2.3. Rotarod Test

Motor function and the coordination of the hindlimbs were examined in a four-lane rotarod unit (Ugo Basile, Comerio, Italy), which consists of 7 cm diameter drums with grooves. Pre-training was performed over three consecutive days. A rod was accelerated from 5 to 10 rpm over a period of 5 min. The rats were given four training trials per day for five consecutive days with an inter-trial interval of 5 min. Lesioned rats were allowed to stay on the drum at a fixed speed of 5 rpm during a one-minute observation period. The latency to fall from the rotating rod was automatically recorded. The test was performed before surgery and 3, 5, 7 and 9 weeks after surgery.

#### 4.2.4. Plantar Test

The plantar test used a standard Ugo Basile test apparatus (Ugo Basile, Comerio, Italy) on curcumin- or vehicle-treated rats. The rat was placed into a transparent acrylic box, and a mobile infrared heating lamp was positioned underneath the targeted hind paw, always in the same defined location. A thermal radiant heat stimulus was then applied to the plantar surface, and the latency of the paw withdrawal response was measured automatically with the help of a photoelectric-sensitive device. The plantar test measures the latency of the withdrawal response of each hind paw. The test was performed before spinal cord surgery and then weekly after SCI. Each paw was stimulated five times. Hyperalgesia was defined as a significant decrease in withdrawal latency.

### 4.3. Immunohistochemical and Histological Staining

Nine weeks after injury, the animals treated with curcumin (*n* = 12) and the control group (*n* = 12) were transcardialy perfused with 4% paraformaldehyde in PBS, and then spinal cords were postfixed overnight and then removed from the spinal canal. The spinal cord was dissected 1 cm cranially and 1 cm caudally from the center of the lesion, mounted in paraffin and cross-sections (5 μm thickness) were cut. To distinguish the gray and white matter, Cresyl violet (0.25 g of cresyl violet dissolved in 100 mL of distilled water with 1 mL of 10% acetic acid) and Luxol-fast blue (1 g of Luxol-fast blue dissolved in 100 mL of 96% ethanol with 5 mL of 10% acetic acid) staining was used. Sections were collected from 1-mm intervals along the cranio-caudal axis from the center of the lesion. Whole images of the spinal cord were taken with an Axioskop 2 plus microscope (Zeiss, Oberkochen, Germany). The areas of remaining grey and white matter within the 2 cm long spinal cord segment and the volume of the cavities were measured using ImageJ software (Wayne Rasband, National Institutes of Health, Bethesda, MD, USA).

For immunohistochemical analysis of axonal sprouting and astrogliosis, primary antibodies against GAP43 (Millipore, Billerica, MA, USA) and GFAP (conjugated with CY3; Sigma, St. Louis, MO, USA) were used. As a secondary antibody, goat anti-mouse IgG conjugated with Alexa-Fluor 488 (Abcam, Bristol, UK) was utilized (for GAP43 staining). A ZEISS AXIO Observer D1 microscope (Carl Zeiss, Weimar, Germany) was used to evaluate the staining results. For graphical processing, Excel (Office 2010, Microsoft, Redmond, WA, USA) and CorelX6 (Corel Corporation, Ottawa, ON, Canada) software were used. In each sample, seven sections were selected at 1-mm intervals on each side along the cranio-caudal axis from the lesion center. For axonal sprouting, the number of Gap43-positive fibers per slide was manually counted. The number of protoplasmic astrocytes per section and GFAP positive area around the lesion cavity were analyzed using ImageJ software (Wayne Rasband, National Institutes of Health, Bethesda, MD, USA).

Separately, staining for NF-κB (p65) in combination with hematoxylin was used to evaluate the NF-κB pathway activity. Animals treated with curcumin (*n* = 23) and control animals (*n* = 20) were transcardially perfused with 4% paraformaldehyde in PBS. Their spinal columns were dissected and left in paraformaldehyde overnight. The next day, the spinal cords were cut 1 cm cranially and 1 cm caudally from the lesion center, then cross-sectioned into 5 μm thick slides mounted in paraffin. Primary rabbit anti-p65 (IgG) antibody (Santa Cruz, Dallas, TX, USA) and secondary goat anti-rabbit IgG conjugated with peroxidase H (Vector Laboratories, Burlingame, CA, USA) were used to stain the cross-sections. The reaction of added DAB (Vector Laboratories, Burlingame, CA, USA) and peroxidase H in the presence of H_2_O_2_ was used to visualize p65 positivity. Sections at 1 mm intervals along the cranio-caudal axis from the lesion were used, and images of the whole cross-sections were taken using a LEICACTR6500 microscope and TissueFAXS software 4.2.6245.1020 (TissueGnostics, Vienna, Austria). HistoQuest analysis software 4.0.4.0154. (TissueGnostics, Vienna, Austria) was used for graphical evaluation of the number of NF-κB (p65)-positive nuclei per mm^2^.

### 4.4. qPCR

Quantitative real-time reverse transcription polymerase chain reaction (qPCR) was used to study the expression of the rat target genes *Sort1* (*Nt-3*), *Fgf2*, *Olig2*, *Gap43*, *Gfap*, *Vegfa*, *Nfkb1*, *Cntf*, *Mip1α* (*Ccl3*), *Rantes* (*Ccl5*), *Cd86*, *Cd163*, *Irf5* and *Mrc1*, 10 and 28 days after SCI induction (in all groups *n* = 4). RNA was isolated from parafomadehyde-fixed frozen tissue sections using the High Pure RNA Paraffin Kit (Roche, Penzberg, Germany), following the manufacturer’s recommendations. A spectrophotometer (NanoPhotometerTM P-Class, Munchen, Germany) was used to quantify RNA amounts. The isolated RNA was reverse transcribed into cDNA using Transcriptor Universal cDNA Master (Roche, Penzberg, Germany) and a thermal cycler (T100™ Thermal Cycler, Bio-Rad, Hercules, CA, USA). The qPCR reactions were performed using a cDNA solution, FastStart Universal Probe Master (Roche, Penzberg, Germany) and TaqMan^®^ Gene Expression Assays (Life Technologies, Carlsbad, CA, USA) (Supplementary Material, Table S1). The qPCR was carried out in a final volume of 10 μL containing 25 ng of extracted RNA. A real-time PCR cycler (StepOnePlus™, Life Technologies, Carlsbad, CA, USA) was used for amplification. All amplifications were run under the same cycling conditions: 2 min at 50 °C, 10 min at 95 °C, followed by 40 cycles of 15 s at 95 °C and 1 min at 60 °C. All samples were run in duplicate, and a negative control was included in each array. Relative quantification of gene expression was determined using the ΔΔ*C*t method [31]. Results were analyzed with StepOnePlus^®^ software (Life Technologies, Life Technologies, Carlsbad, CA, USA). The gene expression levels were normalized using *Gapdh* as a reference gene; control animals with SCI treated with vehicle were used as a calibrator. A log2 scale was used to display the symmetric magnitude for up and down regulated genes.

### 4.5. Cytokine Evaluation

The effects of local and systemic applications of curcumin on the levels of inflammatory cytokines at the center of the spinal cord lesion were determined 1, 3, 7, 10, 14, and 28 days after SCI (curcumin *n* = 5 per time point, vehicle *n* = 5 per time point) according to our previously published method [32]. Two millimeter long segments dissected from the central part of the spinal cord lesion were incubated in cell culture media (DMEM, Sigma, St. Louis, MO, USA) supplemented with 10% FBS and 0.2% primocin. After 24 h of incubation, the media were collected for further investigation. A Customized Milliplex inflammatory cytokine kit (Millipore, Billerica, MA, USA) and Magpix instrumentation software were used to analyze inflammatory cytokines. Rat cytokine Luminex custom eight-plex kits (for IL-2, IL-4, IL-6, IL-8, IL-10, IL-12p70, IFNγ, TNFα, MIP-1α) were used for customized bead assays. The assays were performed in 96-well filter bottom plates according to the manufacturer’s protocol. Antibody conjugated beads were used at a concentration of 5000 beads per marker, again following the manufacturer’s protocol. A biotinylated detection antibody was used with streptavidin-RPE (streptavidin-R-Phycoerythrin) (Life Technologies, Carlsbad, CA, USA) to measure the levels of cytokines on a Luminex xMAP 200 system (Luminex, Madison, WI, USA) and analyzed using Magpix instrumentation software. Mean fluorescence intensity (MFI) was the raw data used to calculate the concentration of each cytokine; a four- or five-parameter logistic fit curve was generated for each cytokine from seven standards.

The lowest standard at least three-times above background was used to determine the lower limit of quantification (LLOQ). The LLOQ was calculated by subtracting the MFI of the background (diluent) from the MFI of the lowest standard concentration and back-calculating the concentration from the standard curve [32].

### 4.6. Statistical Analysis

One-way ANOVA was used to determine the statistical significance of differences between the curcumin and control groups. In the case of repeated measurements (behavioral tests, distribution of effect in the histological and immunohistochemical analyses), two-way repeated measurement ANOVA was used. As a *post hoc* test, the Student-Neuman-Keuls test was utilized (Sigmastat 3.1, Sistat Software Inc., San Jose, CA, USA). If *p* < 0.05 the difference was considered statistically significant. The data was represented as the means ± the standard error of the mean.

## 5. Conclusions

Curcumin, given immediately after SCI, ameliorates the negative impact of injury by improving functional outcome early in the recovery process. The main mechanism of action is via alterations of pro-inflammatory cytokines (reduction of IL-2, TNF-α, and MIP-1α and upregulation of IL-6 and IL-12p70) orchestrated by the reduction of NF-κB activity. Therefore, curcumin has therapeutic potential in the treatment of SCI, particularly at the early stage of lesion development. Curcumin’s therapeutic potential might be reinforced by subsequent therapy to facilitate long-term tissue reconstruction.

## Supplementary Materials

Supplementary materials can be found at http://www.mdpi.com/1422-0067/17/1/49/s1.

## References

[1] Lawrence T. (2009). The nuclear factor NF-κB pathway in inflammation. Cold Spring Harb. Perspect. Biol..

[2] Lin M.S., Lee Y.H., Chiu W.T., Hung K.S. (2011). Curcumin provides neuroprotection after spinal cord injury. J. Surg. Res..

[3] Ormond D.R., Shannon C., Oppenheim J., Zeman R., Das K., Murali R., Jhanwar-Uniyal M. (2014). Stem cell therapy and curcumin synergistically enhance recovery from spinal cord injury. PLoS ONE.

[4] Basso D.M., Beattie M.S., Bresnahan J.C. (1995). A sensitive and reliable locomotor rating scale for open field testing in rats. J. Neurotrauma.

[5] Rossignol S., Schwab M., Schwartz M., Fehlings M.G. (2007). Spinal cord injury: Time to move?. J. Neurosci..

[6] Chen X., Zhou C., Guo J., Sun K., Zhao N., Yang J., Sun Y., Liu X., Hibi T., Liu Z. (2011). Effects of dihydroxylphenyl lactic acid on inflammatory responses in spinal cord injury. Brain Res..

[7] Rafati D.S., Geissler K., Johnson K., Unabia G., Hulsebosch C., Nesic-Taylor O., Perez-Polo J.R. (2008). NF-κB decoy amelioration of spinal cord injury-induced inflammation and behavior outcomes. J. Neurosci. Res..

[8] Aggarwal B.B., Harikumar K.B. (2009). Potential therapeutic effects of curcumin, the anti-inflammatory agent, against neurodegenerative, cardiovascular, pulmonary, metabolic, autoimmune and neoplastic diseases. Int. J. Biochem. Cell Biol..

[9] Begum A.N., Jones M.R., Lim G.P., Morihara T., Kim P., Heath D.D., Rock C.L., Pruitt M.A., Yang F., Hudspeth B. (2008). Curcumin structure-function, bioavailability, and efficacy in models of neuroinflammation and Alzheimer’s disease. J. Pharmacol. Exp. Ther..

[10] Chainani-Wu N. (2003). Safety and anti-inflammatory activity of curcumin: A component of tumeric (*Curcuma longa*). J. Altern. Complement. Med..

[11] Aggarwal B.B., Shishodia S., Takada Y., Banerjee S., Newman R.A., Bueso-Ramos C.E., Price J.E. (2005). Curcumin suppresses the paclitaxel-induced NF-κB pathway in breast cancer cells and inhibits lung metastasis of human breast cancer in nude mice. Clin. Cancer Res..

[12] Ammon H.P., Wahl M.A. (1991). Pharmacology of *Curcuma longa*. Planta Med..

[13] Ormond D.R., Peng H., Zeman R., Das K., Murali R., Jhanwar-Uniyal M. (2012). Recovery from spinal cord injury using naturally occurring antiinflammatory compound curcumin: Laboratory investigation. J. Neurosurg. Spine.

[14] Cao F., Liu T., Xu Y., Xu D., Feng S. (2015). Curcumin inhibits cell proliferation and promotes apoptosis in human osteoclastoma cell through MMP-9, NF-κB and JNK signaling pathways. Int. J. Clin. Exp. Pathol..

[15] Chung S.S., Vadgama J.V. (2015). Curcumin and epigallocatechin gallate inhibit the cancer stem cell phenotype via down-regulation of STAT3-NF-κB signaling. Anticancer Res..

[16] Ma T., Guo C.J., Zhao X., Wu L., Sun S.X., Jin Q.H. (2015). The effect of curcumin on NF-κB expression in rat with lumbar intervertebral disc degeneration. Eur. Rev. Med. Pharmacol. Sci..

[17] Marquardt J.U., Gomez-Quiroz L., Arreguin Camacho L.O., Pinna F., Lee Y.H., Kitade M., Dominguez M.P., Castven D., Breuhahn K., Conner E.A. (2015). Curcumin effectively inhibits oncogenic NF-κB signaling and restrains stemness features in liver cancer. J. Hepatol..

[18] Shishodia S., Potdar P., Gairola C.G., Aggarwal B.B. (2003). Curcumin (diferuloylmethane) down-regulates cigarette smoke-induced NF-κB activation through inhibition of IkappaBalpha kinase in human lung epithelial cells: Correlation with suppression of COX-2, MMP-9 and cyclin D1. Carcinogenesis.

[19] Chen F., Wang H., Xiang X., Yuan J., Chu W., Xue X., Zhu H., Ge H., Zou M., Feng H. (2014). Curcumin increased the differentiation rate of neurons in neural stem cells via wnt signaling *in vitro* study. J. Surg. Res..

[20] Zhu H.T., Bian C., Yuan J.C., Chu W.H., Xiang X., Chen F., Wang C.S., Feng H., Lin J.K. (2014). Curcumin attenuates acute inflammatory injury by inhibiting the TLR4/MyD88/NF-κB signaling pathway in experimental traumatic brain injury. J. Neuroinflamm..

[21] Eggler A.L., Gay K.A., Mesecar A.D. (2008). Molecular mechanisms of natural products in chemoprevention: Induction of cytoprotective enzymes by Nrf2. Mol. Nutr. Food Res..

[22] Arenas-Ramirez N., Woytschak J., Boyman O. (2015). IL-2: Biology, Design and Application. Trends Immunol..

[23] Devi Y.S., DeVine M., DeKuiper J., Ferguson S., Fazleabas A.T. (2015). Inhibition of IL-6 signaling pathway by curcumin in uterine decidual cells. PLoS ONE.

[24] Wang Y.F., Zu J.N., Li J., Chen C., Xi C.Y., Yan J.L. (2014). Curcumin promotes the spinal cord repair via inhibition of glial scar formation and inflammation. Neurosci. Lett..

[25] Yu D.S., Cao Y., Mei X.F., Wang Y.F., Fan Z.K., Wang Y.S., Lv G. (2014). Curcumin improves the integrity of blood-spinal cord barrier after compressive spinal cord injury in rats. J. Neurol. Sci..

[26] Karlstetter M., Lippe E., Walczak Y., Moehle C., Aslanidis A., Mirza M., Langmann T. (2011). Curcumin is a potent modulator of microglial gene expression and migration. J. Neuroinflamm..

[27] Tuttolomondo A., Pecoraro R., di Raimondo D., di Sciacca R., Canino B., Arnao V., Butta C., della Corte V., Maida C., Licata G. (2014). Immune-inflammatory markers and arterial stiffness indexes in subjects with acute ischemic stroke with and without metabolic syndrome. Diabetol. Metab. Syndr..

[28] Dong H.J., Shang C.Z., Peng D.W., Xu J., Xu P.X., Zhan L., Wang P. (2014). Curcumin attenuates ischemia-like injury induced IL-1beta elevation in brain microvascular endothelial cells via inhibiting MAPK pathways and NF-κB activation. Neurol. Sci..

[29] Urdzikova L., Jendelova P., Glogarova K., Burian M., Hajek M., Sykova E. (2006). Transplantation of bone marrow stem cells as well as mobilization by granulocyte-colony stimulating factor promotes recovery after spinal cord injury in rats. J. Neurotrauma.

[30] Goldstein B., Little J.W., Harris R.M. (1997). Axonal sprouting following incomplete spinal cord injury: An experimental model. J. Spinal Cord Med..

[31] Pfaffl M.W. (2001). A new mathematical model for relative quantification in real-time RT-PCR. Nucleic Acids Res..

[32] Urdzikova L.M., Ruzicka J., LaBagnara M., Karova K., Kubinova S., Jirakova K., Murali R., Sykova E., Jhanwar-Uniyal M., Jendelova P. (2014). Human mesenchymal stem cells modulate inflammatory cytokines after spinal cord injury in rat. Int. J. Mol. Sci..

