# Supplementary material for: The Anti-Inflammatory Compound Curcumin Enhances Locomotor and Sensory Recovery after Spinal Cord Injury in Rats by Immunomodulation
Source: Int J Mol Sci. 2015 Dec 31;17(1):49. doi: 10.3390/ijms17010049 (PMC4730294; doi:10.3390/ijms17010049)
Supplement: Supplementary file 1 [file ijms-17-00049-s001.pdf]

Supplementary Materials: The Anti-Inflammatory Compound Curcumin Enhances Locomotor and Sensory Recovery after Spinal Cord Injury in Rats by Immunomodulation

Lucia Machova Urdzikova, Kristyna Karová, Jiri Ruzicka, Anna Kloudova, Craig Shannon, Jana Dubisova, Raj Murali, Sarka Kubinova, Eva Sykova, Meena Jhanwar-Uniyal and Pavla Jendelova

Table S1. TaqMan® Gene Expression Assays.

Gene Name/Gene Symbol	Assay ID
glyceraldehyde 3-phosphate dehydrogenase/Gapdh	Rn01775763_g1
neurotrophin 3/Sort1	Rn01521847_m1
basic fibroblast growth factor/Fgf2	Rn00570809_m1
oligodendrocyte lineage transcription factor 2/Olig2	Rn01767116_m1
growth associated protein 43/Gap43	Rn01474579_m1
glial fibrillary acidic protein/Gfap	Rn00566603_m1
vascular endothelial growth factor A/Vegfa	Rn01511601_m1
nuclear factor of kappa B1/Nfkb1	Rn01399572_m1
ciliary neurotrophic factor/cntf	Rn00755092_m1
macrophage inflammatory protein 1α /Ccl3	Rn01464736_g1
chemokine (C-C motif) ligand 5/RANTES/Ccl5	Rn00579590_m1
CD86	Rn00571654_m1
CD163	Rn01492519_m1
interferon regulatory factor 5/Irf5	Rn01500522_m1
macrophage mannose receptor 1/Mrc1	Rn01487342_m1

Flat Beam Test Categories

(1) 0: The rat is unable to place the affected hind paw on the horizontal surface of the beam, and its hind body falls off the beam in less than 60 s.
(2) 1: The rat is unable to start traversing the beam, but keeps its body on the beam for 60 s.
(3) 2: The rat traverses half of the beam dragging its hindlimbs.
(4) 3: The rat traverses the whole length of the beam dragging its hindlimbs.
(5) 4: The rat traverses the whole length of the beam and one of two hindlimbs is placed on the beam.
(6) 5: The rat traverses the whole length of the beam and places both hindlimbs to aid less than half of its steps.
(7) 6: The rat traverses the whole length of the beam and places both hindlimbs to aid more than half of its steps.
(8) 7: The rat traverses the whole length of the beam with no more than 2 footslips.
